# Congenital Cytomegalovirus Infection as a Cause of Meniere’s Disease in a Young Population: A Case Series

**DOI:** 10.1177/19160216261453902

**Published:** 2026-09-28

**Authors:** Karolina Falkenius-Schmidt, Tatjana Tomanovic, Luca Verrecchia, Johannes Ehinger, Ulrika Löfkvist, Eva Karltorp

**Affiliations:** 1 Department of Otorhinolaryngology, Head and Neck Surgery, Skåne University Hospital, Lund, Sweden; 2 Otorhinolaryngology, Department of Clinical Sciences Lund, Lund University, Sweden; 3 Department of Clinical Science, Intervention and Technology, Karolinska Institutet, Stockholm, Sweden; 4 Medical unit Ear, Nose, Throat, Hearing and Balance, Karolinska University Hospital, Stockholm, Sweden; 5 Mitochondrial Medicine, Department of Clinical Sciences Lund, Lund University, Sweden; 6 Department of Public Health and Caring Sciences, Uppsala University, Sweden

**Keywords:** congenital CMV infection, sensorineural hearing loss, vestibular function, Meniere’s disease, endolymphatic hydrops

## Abstract

**Importance:**

Congenital cytomegalovirus (cCMV) is the most common cause of prenatal infection and the leading infectious cause of sensorineural hearing loss in childhood. Novel data indicate vestibular impairment is common in children with cCMV infection. Associations between endolymphatic hydrops and cCMV infection in humans are under debate, research reports are still scarce.

**Objective:**

To highlight a potential subgroup of Meniere’s disease in young individuals and to support previous findings of recurrent vestibular impairment in children with cCMV infection.

**Design:**

A retrospective descriptive study of a clinical case series.

**Setting:**

Skåne University Hospital, Karolinska University Hospital and Sahlgrenska University Hospital in Sweden.

**Participants:**

Patients with confirmed cCMV infection who presented with symptoms of Meniere’s disease with onset before the age of 25. Patients were identified during routine clinical evaluations.

**Intervention:**

Data were collected from performed vestibular testing, hearing evaluations and imaging results. Vestibular function was assessed with caloric testing and the video head impulse test. Data from medical records were supplemented with updated information obtained from medical interviews.

**Main Outcome Measures:**

cCMV infection with recurrent or newly developed vestibular impairment and sensorineural hearing loss in one or both ears.

**Results:**

Twelve patients aged 15 to 41 years, with confirmed cCMV infection met the inclusion criteria. Six patients experienced the onset of Meniere’s symptoms between 10 and 12 years of age, 4 patients between 16 and 23 years old, and 2 with hearing loss since childhood, developed Meniere’s disease from 30 years of age.

**Conclusion and Relevance:**

cCMV infection may represent a distinct subgroup of endolymphatic hydrops and Meniere’s disease in young individuals. These findings highlight the importance of considering cCMV testing in young patients presenting with symptoms of endolymphatic hydrops.

## Graphical abstract

**Graphical Abstract:**
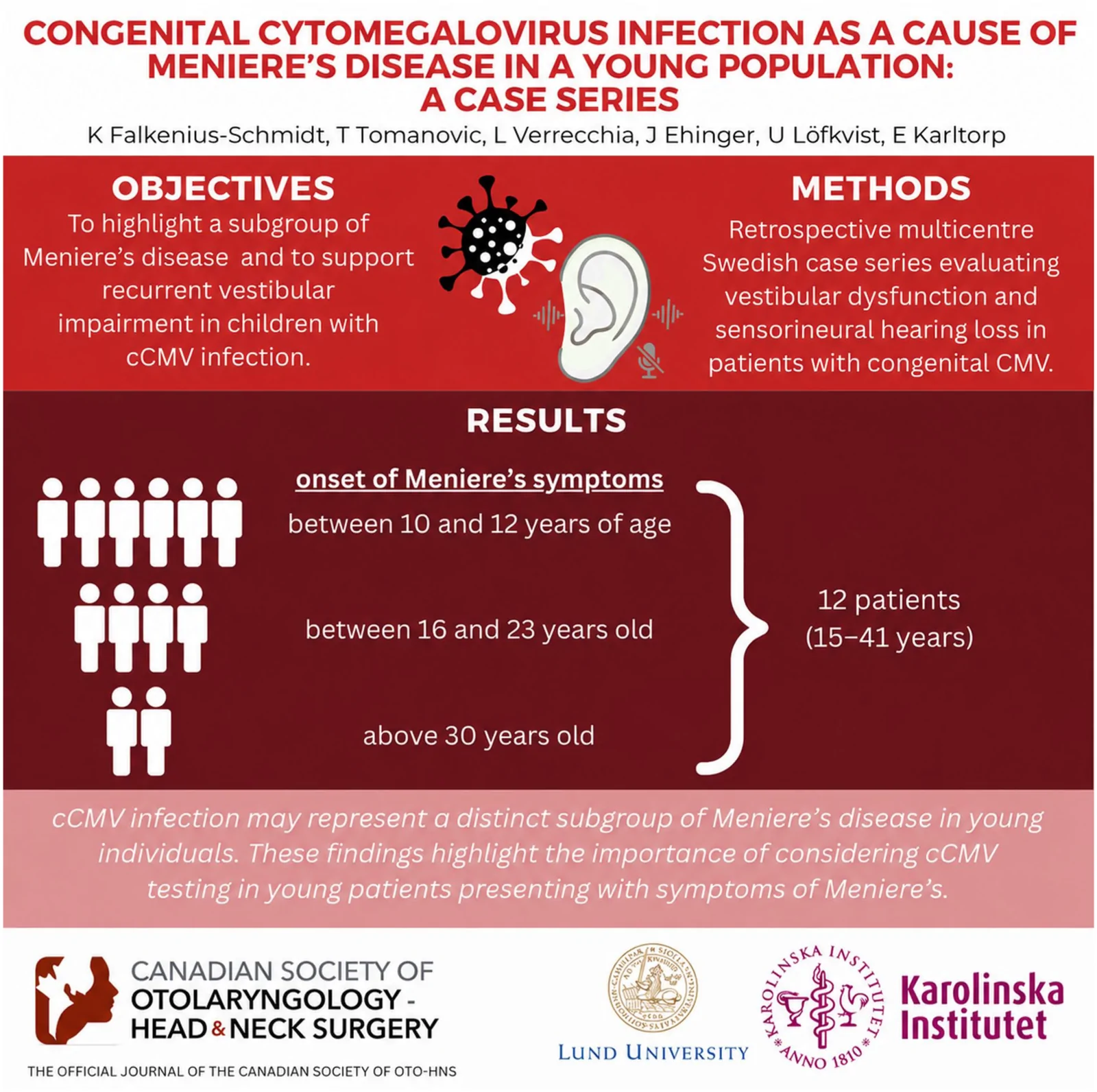
This is a visual representation of the abstract.

## Key Message

- Congenital cytomegalovirus infection may represent a distinct subgroup of endolymphatic hydrops and Meniere’s disease in young individuals.

## Introduction

Congenital cytomegalovirus (cCMV) is the most common cause of congenital infection, with an average birth prevalence of 0.64%.^1,2^ It is a major cause of neurodevelopmental abnormalities and the leading infectious cause of sensorineural hearing loss (SNHL) in childhood.^3^ Children born with the infection might express symptoms at birth, indicating clinical manifestations of the infection that allow for early diagnosis. However, about 90% of the children are asymptomatic at birth,^4^ making detection challenging as diagnosis requires a sample of body fluid (blood, urine, or saliva) collected within the first 2 to 3 weeks of life, to distinguish congenital infection from a more harmless postnatal infection.^5^ Both symptomatic and asymptomatic cCMV infections at birth may lead to lifelong sequelae, including progressive hearing loss and developmental delays.^6^ Due to limited experience of universal newborn CMV screening and long-term follow-up, understanding of the full impact of the infection remains limited.

Lately there has been attention on vestibular symptoms in this population, showing that vestibular impairment is common among children with cCMV infection and SNHL as expression of a particular viral influence on the inner ear.^7,8^ Vestibular impairment has been described in children with cCMV infection without hearing loss, indicating vestibular deficits might be more frequent than hearing loss among cCMV-infected individuals.^7,9-11^ Progress in magnetic resonance imaging (MRI) techniques has enabled visualization of endolymphatic hydrops (EH) in living human subjects.^12^ Recently, Kahn et al assessed 6 cases of young individuals with cCMV infection and audio-vestibular sequelae by describing inner ear anatomy after MRI and signs of endolymphatic pressure disorder.^13^

Early histopathological studies have revealed EH in all patients developing clinical syndrome of MD.^14-16^ MD is a multifactorial disorder, most consistent associations are found between migraine and immune-related disorders. MD can have unilateral or bilateral presentation. Although bilateral MD is less common, it is more frequently associated with a family history of vertigo. Recent genetic research supports both autosomal dominant and recessive patterns of inheritance.^17^ MD is considered unusual among younger individuals, and especially among children.^18^

Committee of the Bárány Society has published consensus in diagnostic criteria for MD.^19^ MD is characterized by repeated vertigo attacks lasting longer than 20 minutes, accompanied by tinnitus and/or fullness in the ear with SNHL, typically affecting lower frequencies on the audiogram.

To identify subgroups of MD, a multicenter study investigating clinical phenotypes in patients with unilateral MD was published in 2017.^20^ Five subgroups were identified: (1) sporadic classic MD (53%), (2) MD associated with migraine (15%), (3) familial MD (13%), (4) MD accompanied by autoimmune disorders (11%), and (5) delayed MD with debut of vertigo months or years after the onset of hearing loss (8%).

To our knowledge, cohort studies and larger case series examining potential associations between MD and cCMV infection in humans are scarce. Improved understanding of this potential subgroup of MD is therefore desirable. This study presents 12 clinical cases of young individuals with cCMV infection exhibiting inner ear symptoms resembling MD.

## Methods

### Participants

All cases were identified at 3 tertiary referral centers for otorhinolaryngology in Sweden (Skåne University Hospital, Karolinska University Hospital and Sahlgrenska University Hospital). The inclusion criteria required confirmed cCMV infection together with recurrent or newly developed vestibular symptoms according to the Bárány Society criteria and/or SNHL in one or both ears, with symptom onset before 25 years of age. No systematic selection was applied.

### Data Collection

Ethical approval (Dnr: 2018/394) and informed consent were obtained. Details of patient history were collected from medical records and medical interviews conducted according to a study protocol (K.F.-S., E.K., T.T.). Background characteristics that might be related to inner ear disorder and EH were documented, such as previous medical history, self-perceived hearing difficulties, tinnitus, noise exposure, heredity for MD, vestibular symptoms, childhood balance issues, walking age, and history of migraine. Retrospective data were also collected from performed vestibular testing, hearing evaluations and imaging results.

### cCMV Diagnosis

Congenital CMV infection was confirmed by polymerase chain reaction (PCR) detection of CMV DNA from dried blood spot (DBS) samples collected at birth, or from CMV DNA analysis of neonatal body fluids.

### Vestibular Assessment

Vestibular function was assessed with caloric testing and the video head impulse test (v-HIT). The relationship between these 2 tests was investigated, since they might be a marker for MD.^21^

### Caloric Test

Bilateral caloric stimulation was performed to evaluate function in the lateral semicircular canal by using bithermal water stimulation. The velocity peak of caloric nystagmus is considered to be the caloric response where a caloric ratio (CR) ≥0.2 (20% difference) was considered pathological.^22^

### Video Head Impulse Test

The vestibule ocular reflex (VOR) stabilizes images on retinas of the eye during head movement. Information regarding changes in the VOR of all 6 semicircular canals in high frequency range of head movements is evaluated with v-HIT. It was performed in the standard manner with either Synapsys^®^ or ICS^®^ Impulse’s devices. All head impulses were completed by experienced practitioners. Gain and presence of corrective saccade are the outcome parameters for head impulses. Pathological range for lateral semicircular canal is <0.8 and for vertical canals <0.7.

### Hearing Evaluation

Since 2007, Sweden has implemented universal newborn hearing screening using otoacoustic emissions (OAE), but some university hospitals began already in the mid-1990s. Infants who fail the OAE test are evaluated with standardized protocol.^23^ Before OAE testing was established in Sweden, hearing evaluation in children was performed at 10 to 12 months and 4 years of age, respectively. Depending on the child’s age and ability to actively participate, a pure tone audiogram may also be conducted in various clinical settings. The pure tone average (PTA) is calculated as the average of hearing threshold levels at 4 key frequencies: 500, 1000, 2000, and 4000 Hz.

### Imaging Data

The first visualization of EH was accomplished in 2010, using a high-sensitivity heavily T2-weighted 3D-FLAIR sequence at 3T. Technical development has resulted in further imaging of the different spaces on MRI with protocol referred “HYDROPS” (Hybrid of the reversed image of the positive endolymph signal and native image of the positive perilymph signal) since 2013.^12^

Besides imaging of EH, the patients were examined with brain MRI to assess possible changes in the brain parenchyma. In cases of cCMV infection, the most specific imaging findings are temporal lobe lesions, but other abnormalities such as ventriculomegaly, cortical malformations, and calcifications may also be noticed.^24^

## Results

A case series of 12 patients with confirmed cCMV infection and MD were identified. Five patients were older than 25 at inclusion but reported symptom onset (hearing loss and/or vertigo) at a younger age. Ten of the 12 patients had cCMV confirmed by PCR analysis of DBS samples, while 2 were diagnosed by CMV DNA analysis in neonatal body fluids.

All patients reported symptoms resembling MD, objectively confirmed through hearing evaluations, vestibular testing, and/or MRI. An overview of all cases is presented in Table 1. The combination of outcome in caloric test and v-HIT could be evaluated in 10 of the patients, one had not undergone caloric test (Case 8), and one was not investigated with v-HIT (Case 3) (Table 2). Ten patients received medical treatment due to fluctuating/ progressive SNHL or vertigo (Table 3).

**Table 1.** Demographics, Hearing and Vestibular Outcome in Individuals With cCMV Infection and Meniere’s Symptoms (n = 12).

Case	Sex M/F	Age at cCMV diagnosis (y)	Hearing in early childhood (<7 y)	Hearing at time of study	Age at onset of vertigo (y)	Vestibular episodes	Caloric response R/L	v-HIT ant. R/L	v-HIT lat. R/L	v-HIT post. R/L	MRI Brain	MRI HYDROPS	Motor development
**1**	F	22	**R profound** L normal	**R profound** L normal	11	3	**R** ↓ L normal	R 0.9 L 0.9	R 0.9 L 0.9	R 0.9 L 0.9	Normal	X	Normal
**2**	M	21	**R profound** **L mild**	**R profound** **L profound**	12	>3	**R** ↓ **L** ↓	R 0.9 L 0.9	R 0.9 L 0.9	R 0.9 L 0.9	Normal	X	**Delayed (16 months)**
**3**	M	14	**R moderate** L normal	**R profound** **L moderate**	10	>3	**R** ↓ **L 0**	X	X	X	Normal	X	**Atypical**
**4**	F	14	Passed newborn screening	R normal **L moderate**	12	2	R normal **L 0**	R 0.9 L 0.9	R 0.9 L 0.9	R 0.9 L 0.9	**WML**	X	Normal
**5**	F	31	R normal **L profound**	**R mild** **L profound**	30	2	**R 0** L normal	R 0.9 L 0.9	R 1.0 **0.6** L	R 0.8 **0.6** L	X	Hydrops * R + L	Unknown
**6**	M	9 months	R normal **L profound**	R normal **L profound**	16	>3	**R 0** **L 0**	**R 0** **L 0**	**R 0** **L 0**	**R 0** **L 0**	Normal	X	**Delayed (22 months)**
**7**	M	Neonatal	R normal L normal	**R moderate** **L moderate**	11	2 drop attacks	**R 0** **L 0**	**R 0.5** **L 0.6**	**R 0.7** L 0.9	**R 0.4** **L 0.7**	**WML**	No	**Delayed (24 months)**
**8**	F	Neonatal	R normal L normal	**R mild** **L moderate**	20	>3	X	R 0.9 L 0.9	R 0.9 L 0.8	R 0.8 L 0.8	**WML**	Hydrops * L	Normal
**9**	F	32	R normal **L profound**	**R moderate** **L profound**	22	>5	R normal **L 0**	R 0.8 **L 0.5**	R 0.9 **L 0.6**	R 0.8 **L 0.5**	Normal	Hydrops * R + L	Normal
**10**	M	38	**R mild** L normal	**R mild** **L severe**	35	>3	**R 0** **L 0**	R 0.7 **L 0.6**	R 0.9 **L 0.5**	**R 0.4** **L 0.5**	X	X	Normal
**11**	M	28	**R profound** L normal	**R profound** L normal	23	>5	**R** ↓ **L 0**	R 0.9 L 0.9	R 0.9 **L 0.3**	R 0.7 **L 0.5**	**WML**	Hydrops * R + L	Normal
**12**	F	21	**R severe** L normal	**R severe** **L moderate**	12	>3	**R** ↓ **L** ↓	R 0.9 L 0.8	R 0.8 L 0.9	R 0.8 L 0.8	**WML**	Hydrops * L	Normal

Abbreviations: M, Male; F, Female; cCMV, congenital cytomegalovirus infection; R, Right; L, Left; v-HIT, video head impulse test; ant./lat./post., anterior/lateral/posterior semicircular canal; MRI, Magnetic Resonance Imaging; WML, White Matter Lesions; Normal, no findings of WML.

Additional findings as described in medical records and interviews. Pathologic results are highlighted with bold characters.

Hearing, hearing loss according to WHO classification; mild 26 to 40 dB, moderate 41 to 60 dB, severe 61 to 80 dB, profound ≥81 dB.

Motor development, walking age without aid ≥16 months is here considered delayed, atypical if history of problems with coordination/balance in ordinary tasks in everyday life.

↓: Depressed caloric function; 0: No/Absent caloric function; X: Not done/no data.

* MRI with hydrops protocol.

**Table 2.** The Combination of Results in Caloric Test and v-HIT for 20 Ears in 10 of 12 Reported Cases (Cases 3 and 8 Not Included).

Caloric test normal + v-HIT normal	15%
Caloric test normal + v-HIT abnormal	5%
Caloric test abnormal + v-HIT abnormal	40%
Caloric test abnormal + v-HIT normal	40%

Abbreviation: v-HIT, video head impulse test.

**Table 3.** Medical Treatments Documented in Medical Records Among Individuals With cCMV Infection and Meniere’s Symptoms (n = 12).

Case	Steroids (progressive hearing loss)	Motion sickness medicine (vertigo/nausea)
Case 1	Oral	Diemenhydrinate + Cinnarzine
Case 2	Oral	No
Case 3	No	No
Case 4	No	Promethazine
Case 5	Oral + intratympanic	No
Case 6	No	No
Case 7	Oral	Promethazine
Case 8	Oral	No
Case 9	Oral + intratympanic	No
Case 10	No	Betahistine
Case 11	No	Betahistine + Promethazine
Case 12	No	Dimenhydrinate + Cinnarzine

Abbreviations: cCMV, congenital cytomegalovirus; No, no treatment offered.

### Case 1

Twenty-two-year-old woman with profound unilateral SNHL (PTA >85 dB) in right ear, diagnosed at 6 years of age. Her motor skill development was normal. At the age of 11, she had a sudden episode of vertigo, nausea and vomiting. At age 20, similar symptoms reoccurred. During episodes of vertigo, she noticed changes in hearing quality, which established a definite MD diagnosis.

PTA during a symptom-free period revealed stable profound SNHL in the right ear and normal hearing in the left. MRI of the temporal bone confirmed normal anatomy. Vestibular examination showed normal results for v-HIT and significantly reduced caloric function in the right ear. Due to suspicion of possible underlying cCMV infection, this diagnosis was ultimately confirmed through the DBS analysis.

### Case 2

Twenty-one-year-old man diagnosed with unilateral hearing loss in the right ear during newborn screening test. Profound hearing loss was confirmed at age 7 (PTA >103 dB). By this time, left ear also showed mild hearing loss (PTA 34 dB). Delayed achievement of gross motor milestones, including independent sitting and walking, was observed during early childhood. At the age of 12, he experienced a severe vertigo attack accompanied by increased tinnitus and sudden hearing loss in his left ear which resembled a definite MD. Over the subsequent years, he had recurrent similar episodes and the hearing loss in his left ear progressed. Vestibular testing revealed reduced caloric function in both ears but normal v-HIT. MRI showed normal inner ear anatomy. Further evaluation with DBS analysis confirmed cCMV infection. The patient underwent cochlear implant surgery in the left ear.

### Case 3

Nineteen-year-old man diagnosed with unilateral deafness in his right ear at 2 years of age. At age 10, hearing in the left ear began fluctuating and gradually deteriorate. Gross motor development was atypical, with difficulties in balance- and coordination-dependent activities. When hearing in the left ear started to deteriorate, his balance also worsened. From age 10, he has experienced symptoms of definite MD. These symptoms typically last between one to several hours and are often triggered by stress and anxiety. He has no history of migraine. Over the years, he has undergone multiple investigations, including audiograms, vestibular testing, normal MRI and lumbar puncture. The tests have consistently shown depressed caloric response in the deaf right ear and no caloric response in the fluctuating left ear. The diagnosis of cCMV infection was confirmed when he was 14 years old. At age 15, he received a cochlear implant in the right ear.

### Case 4

Fifteen-year-old girl who passed neonatal hearing screening on both sides and had normal motor development in early childhood. When 12 years old, she had attacks with severe headache and dizziness, and MRI revealed bilateral supratentorial white matter defects. The intense headaches declined and 1 year later she experienced tinnitus and hearing fluctuations in the left ear (PTA 24 dB) and normal hearing in the right side. A few months later, she experienced severe episodes of vertigo, nausea and vomiting referring to definite MD. Vestibular testing showed no caloric response and preserved v-HIT gain on the left side. Audio vestibular symptoms together with white matter defects on MRI lead to suspicion of cCMV infection, subsequently confirmed through DBS analysis. Further clinical evaluation at age 15 showed unchanged vestibular results but a progressive hearing loss in left side (PTA 43 dB).

### Case 5

Thirty-one-year-old woman was diagnosed with single-sided deafness in her left ear during early childhood. At age 30, she experienced 2 recurrent episodes of vertigo, occurring 2 months apart. Afterwards, she had additional attacks accompanied by sensation of fullness and fluctuating hearing levels in her previously normal ear. Clinical evaluation raised suspicion of definite MD. Caloric test revealed normal reaction on the left side but no caloric response was noticed on the right side where evaluation with v-HIT was normal for all 3 semicircular canals. MRI HYDROPS revealed bilateral EH in the inner ear, confirming MD (Figure 1). The patient has a family history of MD; however, a recent DBS analysis confirmed cCMV infection.

**Figure 1.**
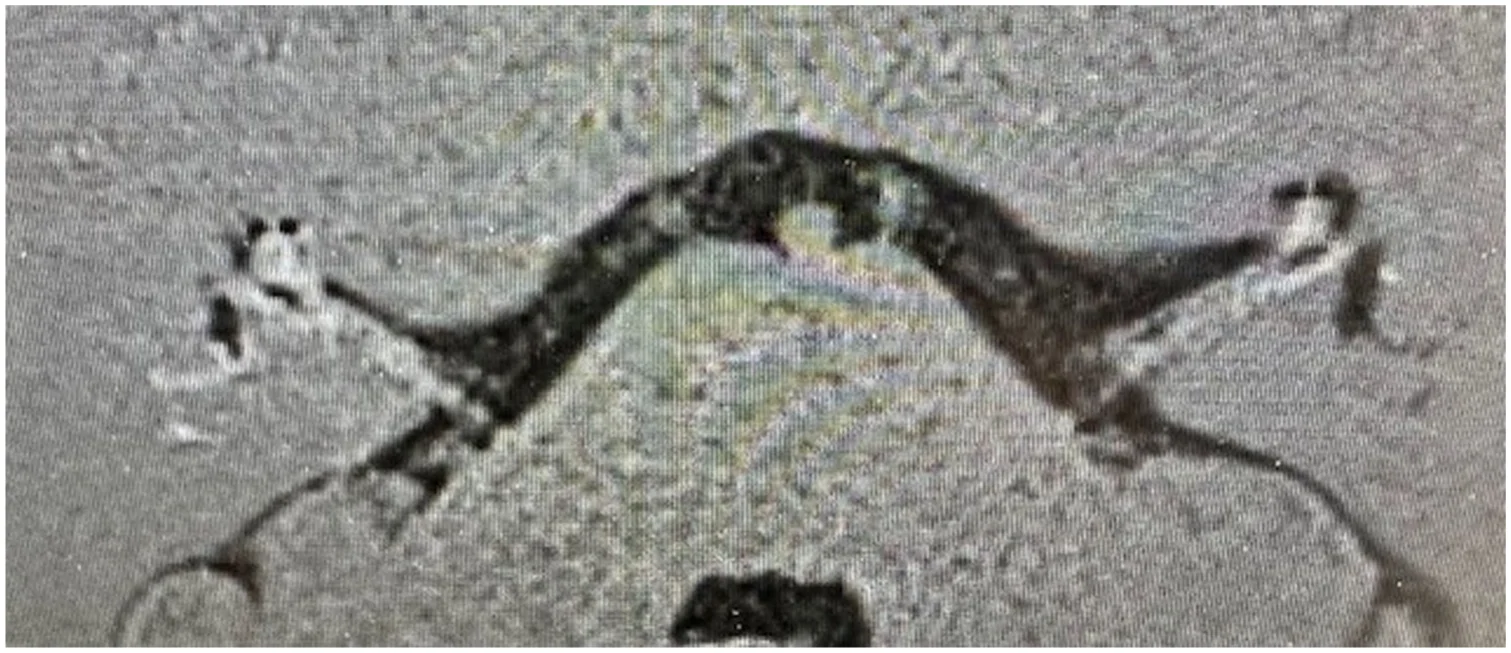
MRI HYDROPS of case 5, showing bilateral endolymphatic hydrops in the inner ear.

### Case 6

Twenty-two-year-old man with confirmed cCMV infection, diagnosed in infancy following delayed achievement of age-appropriate gross motor milestones, was otherwise initially asymptomatic. However, left-sided deafness was identified in early childhood. Vestibular testing with caloric test and v-HIT at age 12 showed no vestibular function. At 16 years of age, he experienced fluctuating hearing loss in his better ear, accompanied by increased tinnitus and a sensation of fullness. Audiogram showed intermittent low-frequency hearing loss in the right side. He reported no episodes of vertigo, which can be explained by the confirmed absence of vestibular function in both ears. MRI of the brain was normal. Symptoms of fluctuating hearing loss and intense tinnitus have reoccurred many times per year since onset, lasting from a couple of weeks to 3 months. These symptoms have been considered consistent with probable MD, despite absence of vertigo. To compensate for the severe hearing deficit during episodes of suspected hydrops, he received a cochlear implant in his deaf ear at age 17.

### Case 7

Nineteen-year-old man with neonatally confirmed cCMV infection detected during routine testing at neonatal care unit prompted by early oxygenation problems. He was initially considered asymptomatic but later showed delayed motor development. Hearing was normal in early childhood, but at 8 years of age, it began to fluctuate in his left ear. At age 11, he experienced a sudden vertigo, nausea and a drop attack. These symptoms reoccurred several times. By age 15, hearing in his other ear also started to fluctuate. At age 18, vestibular evaluation showed no reaction on caloric test and v-HIT showed decreased function on both sides. Given the symptoms and clinical findings, he was diagnosed with bilateral definite MD. MRI of the brain showed multiple white matter lesions, consistent with cCMV infection. Over recent years, his hearing has progressively deteriorated to profound levels in both ears, and he has received bilateral cochlear implants.

### Case 8

Forty-one-year-old woman with neonatally confirmed cCMV infection due to established maternal infection and characteristic white matter lesions on MRI of the brain, but initially without hearing loss or other specific symptoms. At age 20, she began experiencing intermittent fullness sensation and fluctuating hearing levels in her left ear. Ten years later, similar symptoms occurred in the right ear, together with tinnitus, vertigo, nausea and vomiting. These symptoms recurred several times over the next 4 years, lasting a few hours, and definite MD was confirmed. V-HIT was normal. Audiogram showed PTA 58 dB on left side, 28 dB on the right. She has not experienced any similar episodes during the past 6 years. Her hearing remains stable, although she is dependent on hearing aids on both ears. Evaluation by using MRI HYDROPS confirmed cochlear and vestibular EH on both sides, but more distinct findings on the left.

### Case 9

Thirty-two-year-old woman diagnosed at the age of 4 with left-sided deafness. She had normal motor development during early childhood. Hearing in the right side began to fluctuate at 10 years of age. At 22 years, she awoke with severe hearing loss in her right ear, accompanied by dizziness. Her hearing in the right ear later stabilized at a moderate level (PTA 48 dB). She had normal vestibular function until the age of 31, when she lost the function in left side in an acute vestibular episode. By age 32, she again experienced intense vertigo episodes with fluctuating hearing loss in the right ear. Caloric testing and v-HIT showed loss of function on the left side but still normal reaction on the right side. Vertigo episodes with fluctuating hearing loss in the right ear recurred multiple times, leading to a suspicion of definite MD. Further evaluation using MRI HYDROPS showed cochlear and vestibular EH bilateral. A retrospective DBS analysis confirmed cCMV infection. She has now received a cochlear implant in the left ear.

### Case 10

Forty-year-old man with a diagnosed mild hearing loss (PTA 25 dB) in the right ear in early childhood, otherwise healthy. Some family members had a history of right-sided hearing loss. At age 35, he suddenly experienced recurrent vertigo attacks typical for definite MD, but initially without any hearing sensation. The left ear gradually started to fluctuate, together with ear fullness and tinnitus. Evaluation at age of 38 revealed vestibular areflexia in caloric test on both sides. V-HIT showed gain asymmetry for lateral canal, with a lower function in the left side. Vertical gain on both sides were decreased without any side difference. He suffered from recurrent vertigo and dizziness, along with fluctuating hearing loss in the left ear over a 5-year period. A retrospective DBS analysis confirmed cCMV infection. The most recent hearing evaluation showed PTA 28 dB in the right ear, 68 dB in the left ear.

### Case 11

Twenty-nine-year-old man with profound unilateral right hearing loss diagnosed at the age of 5. Normal motor development during childhood. At the age of 23 he had his first episode of acute vestibular syndrome, with nausea, vomiting, persistent rotation vertigo and incapacity to stand up, spontaneously resolved after a few days. Vertigo spells recurred sporadically. At age 28, vestibular evaluation showed low caloric response on the left side and v-HIT significant gain reduction on the left lateral and posterior canal, contralaterally to the ear with severe hearing loss (Figure 2). Presence of crossed hearing/vestibular damage and a VOR discrepancy between caloric responses and v-HIT, led to further investigation. Neither a definitive nor a probable MD could initially be formulated, however the recurrent symptomatic episodes made MD highly suspected. MRI revealed unspecific white matter lesions, and MRI HYDROPS showed endolymphatic atelectasis of the left vestibulum together with significant EH of the vestibulum on the right side. DBS analysis confirmed cCMV infection.

**Figure 2.**
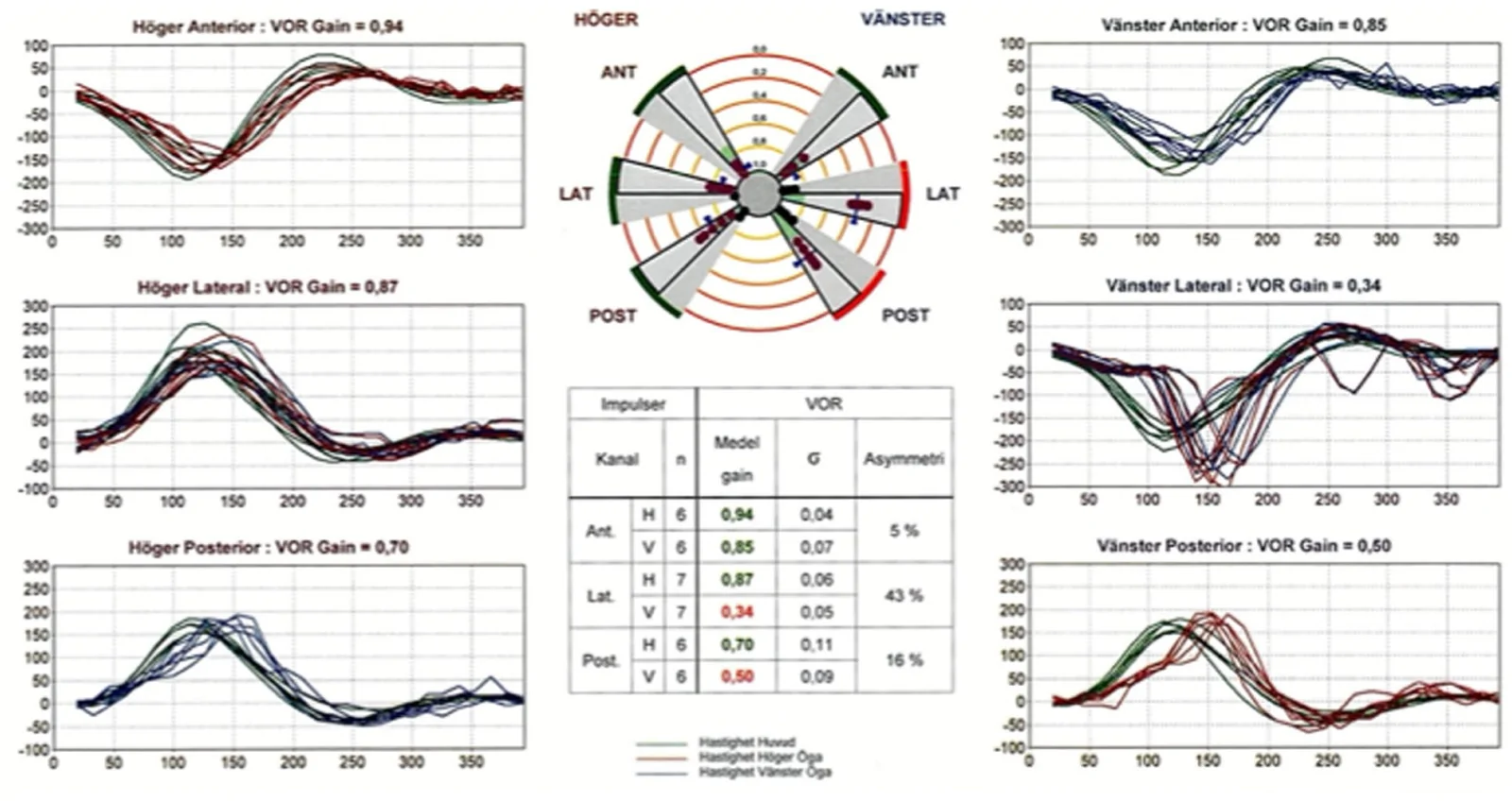
v-HIT showing VOR gain reduction for left lateral and left posterior canals with the presence of consequent cover saccades. “Vänster” imply left side, “höger” the right side. VOR, vestibule ocular reflex; v-HIT, video head impulse test.

### Case 12

Twenty-two-year-old woman diagnosed with severe hearing loss (PTA 68 dB) in her right ear at the age of 3. Her motor skill development was normal. When 8, hearing in her better ear began to fluctuate, and she started to complain on periods with headaches. By nine, she began having episodes of dizziness, accompanied by unsteadiness and intolerance to head movements. During adolescence, she experienced occasional vertigo attacks accompanied by tinnitus and nausea, resembling a definite MD. Over the years, she has undergone multiple investigations, including audiograms, vestibular testing, and MRI. The caloric tests have shown depressed caloric results on both sides. Review of a previously performed MRI revealed white matter lesions compatible with cCMV infection. The diagnosis was finally confirmed through DBS analysis at age of 21. A supplementary MRI HYDROPS showed significant EH on the left side.

## Discussion

Since the discovery that inoculation of CMV into the endolymphatic sac in animals can induce inner ear hydrops, it has been hypothesized that CMV may play a role in the pathogenesis of MD.^25,26^ The cases of cCMV infection and MD presented here provide clinical support for this hypothesis, at least within a young population. A research group from the Netherlands described episodic vestibular symptoms in 5 paediatric cases with cCMV infection.^27^ Although diagnosing classical MD symptoms in children can be challenging, their findings align with ours, further supporting a potential link between cCMV infection and MD. Young patients with MD are rare in the general population, and few publications have focused on this group. Although the incidence remains uncertain, cCMV infection may represent a subgroup of MD in young individuals.

In this case series, it is notable that hearing loss developed long before onset of vertigo. According to the 2015 classification,^28^ a clinical presentation where vertigo and hearing loss do not occur simultaneously is known as delayed MD. The presence of EH further supports the diagnosis of MD. Our case study describes a phenotype with delayed MD related to cCMV infection.

Although all the described cases have cCMV infection and Meniere’s symptoms in common, they exhibit a range of various clinical manifestations and differ from each other. The pathophysiology of MD is complex and heterogeneous, with multiple potential causes stemming from various underlying conditions. There is currently no known biological marker for the diagnosis and prognosis of MD. One potential diagnostic marker is dissociation between caloric test results and head impulse test findings. Among MD patients, the most common pattern is an abnormal caloric response with a normal v-HIT.^21^ In this study, several of the cases expressed both abnormal caloric response and abnormal v-HIT. Hypothetically, discrepancies between these 2 tests might be related to the extent of damage to type 1 and type 2 vestibular hair cells. The mechanism behind inner hair cell damage in cCMV infection remains unclear, especially in relation to delayed symptoms. Most of the patients suffered from severe inner ear deterioration combined with hydrops. In many cases, progressive hearing loss led to cochlear implantation. The inner ear impairment observed in this group may be more severe compared to other subgroups of MD.

Many of the described patients underwent a prolonged series of investigations before a correct understanding of their symptoms. Most of them had normal inner ear anatomy on MRI scans performed without a specific hydrops protocol. It is likely that MRI conducted with HYDROPS protocol reveals EH, as was seen in 5 of the 12 study patients.

cCMV infection has not traditionally been considered a potential differential diagnosis in patients with sudden SNHL and/or episodic vestibular symptoms. The advent of PCR technology enables retrospective diagnosis of cCMV infection in DBS cards from newborns, but can only be performed in countries where DBS have been stored. Sweden has a unique history of a DBS register since 1975, facilitating retrospective evaluation of cCMV infection in patients with MD.

In conclusion, this study underscores the importance of further cCMV testing for patients presenting with symptoms of MD, especially when hearing loss precedes vestibular symptoms. It also advocates for long-term storage of DBS from birth. Identifying cCMV infection in young patients with MD could pave the way for additional research, new treatment options, and, most importantly, raise awareness of cCMV infection as a distinct subgroup within the broader group of patients with EH.
